# A Description of Two Cases of Non-Contagious Molluscum, or Hypertrophy of the Cellular Tissue

**Published:** 1851-12

**Authors:** Henry P. Ritter

**Affiliations:** Washington, D. C.


ART. III. —*A Description of Two Cases of Non-contagious Molluscum, or Hypertrophy of the Cellular Tissue.* By HENRY P. RITTER, M. D., of Washington, D. C.

During the winter of 1850, the following interesting cases came under my observation:

The first was that of a mulatto woman, who had died from phthisis pulmonalis, aged twenty-four, of diminutive stature, and much emaciated. Her whole person, with the exception of the face and feet, was completely studded with tubercles, varying in size from a pin's head to that of a large hen's egg. Those situated upon the scalp were somewhat different from those on the body and extremities. They were more pendulous and attached merely by small pedicles or necks, to the surface. One very large excrescence, situated immediately behind the right ear, had this form in a remarkable degree. Its neck was not larger in circumference than a crow-quill, and it appeared as if very little violence would suffice to detach it altogether from its connection. Those situated upon the body and extremities, were not so large, but were much more numerous, and had larger surfaces of attachment. Those on the hands were possessed of greater density, and somewhat more flattened, owing, probably, to the pressure to which they were constantly subjected. In other respects they resembled those situated upon the unexposed parts of the person. In fact, as I have just remarked, there was not a square inch of cuticle which was not covered with these tumors, with the exception of the face and soles of the feet, although the lower extremities were more free from them than the trunk or the upper extremities.

The surface of the subject might be not unaptly compared to that of the scrub-oak, with this difference, that whilst the excrescences of the latter presented themselves as rough, incompressible, irregular uodules, those of the former were pear-shaped, smooth tumors, possessing, at the same time, a degree of elasticity and denseness, as if consisting of caoutchouc. Upon incision they were found to consist of cellular tissue and fatty matter. Blood vessels, of considerable size, were distinctly visible in the larger tumors, but there was no softening or breaking down observable in any of them.

I was unable for some time to learn much of the previous history of this case; but in February of the same winter, a brother of the deceased presented himself at the infirmary of this city, to be treated for paronychia, which had commenced in the thumb of the right hand, and extended up the forearm, nearly to the elbow joint. Incisions were made, and large quantities of pus evacuated, considerable sloughing ensued, and after the loss of his thumb, by amputation, he made a speedy recovery.

He was a strong robust man, of about twenty-nine years of age, and informed me that his health had always been excellent, never having suffered from a day's indisposition, with the exception of the attack of paronychia just mentioned. His intellect was not very acute, although he was far from being idiotic. He had been affected with these excrescences since the age of seven months. Some time previous to his birth, his mother was attacked with this eruption, and though she had borne a number of children, none of them were affected except the brother and sister, whose cases are here described. These were the two youngest children, and were born after the mother became the subject of this disease.

The eruption commenced in the shape of small papules, which continued slowly to increase in size and numbers, up to the present time. His lower extremities are comparatively free from them, but not perfectly so. They caused him but little uneasiness, except when heated, or in a state of perspiration, when they produced a most intolerable itching, but which was much allayed by bathing in cold water or spirits.

He frequently scratched off these tubercles when small, but the abraded surface soon healed, and another crop was shortly reproduced.

His sister was free from the disease till she was fifteen years of age, when the eruption made its appearance precisely as in the case of her brother, but progressing with greater rapidity, the excrescences being more numerous, and attaining a much greater size.

The mother died some ten or twelve years ago, but I have never been able to procure a reliable description of her case, or learn the cause of her death.

